# Supplementary material for: Hypothalamic astrocyte NAD+ salvage pathway mediates the coupling of dietary fat overconsumption in a mouse model of obesity
Source: Nat Commun. 2024 Mar 7;15:2102. doi: 10.1038/s41467-024-46009-0 (PMC10920699; doi:10.1038/s41467-024-46009-0)
Supplement: Supplementary file 1 — Supplementary Information [file 41467_2024_46009_MOESM1_ESM.pdf]

Supplementary Information

Hypothalamic astrocyte NAD^+ salvage pathway mediates the coupling of dietary fat overconsumption to obesity in mice

Jae Woo Park[1], Se Eun Park[1], Wuhyun Koh[2], Won Hee Jang[1], Jong Han Choi[3], Eun Roh[4], Gil Myoung Kang[5], Seong Jun Kim[1], Hyo Sun Lim[1], Chae Beom Park[1], So Yeon Jeong[1], Sang Yun Moon[1], Chan Hee Lee[6], Sang Yeob Kim[7], Hyung Jin Choi[8], Se Hee Min[5,9], C. Justin Lee[2], Min-Seon Kim[5,9]

* Correspondence and requests for materials should be addressed to M.S.K (mskim@amc.seoul.kr).

This PDF file includes:

Supplementary Figure 1-9

Supplementary Table 1

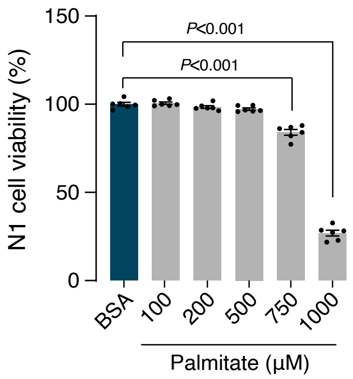

Supplementary Figure 1. Effect of palmitate treatment on neuronal cell viability

N1 hypothalamic neuron cells were treated with either 5 mM BSA solution or palmitate at the indicated concentrations dissolved in 5 mM BSA solution for 48 h (n = 6 wells).

One-way ANOVA followed by Fisher's LSD test. Two independent replicates were performed.

Results are presented as mean ± SEM. Source data are provided as a Source Data file.

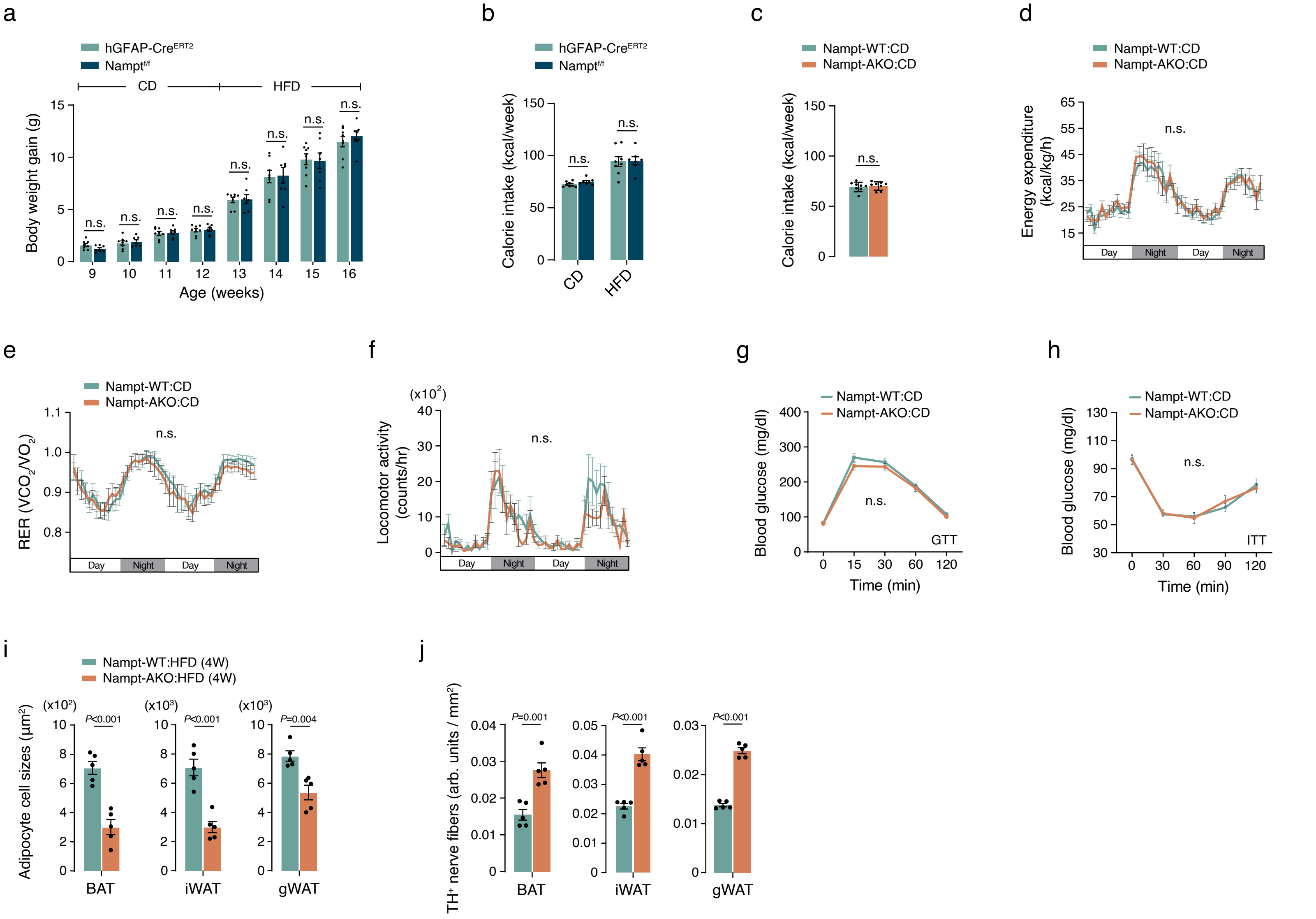

Supplementary Figure 2. Metabolic phenotype analysis in astrocyte-specific Nampt knockout male mice

a, **b** Comparison of body weights (**a**) and calorie intakes (**b**) between hGFAP-Cre^{ERT2} and Nampt$^{f/f}$ male mice following tamoxifen injection during sequential chow diet (CD) and high-fat diet (HFD) feeding conditions (hGFAP-Cre^{ERT2}, n = 8 mice; Nampt$^{f/f}$, n = 7 mice). **c–h** Calorie intakes (n = 8 mice), energy expenditure (n = 8 mice), RER (n = 8 mice), and locomotor activity (n = 6 mice) in 16-week-old Nampt-AKO and Nampt-WT male mice fed a CD. **g**, **h** Glucose and insulin tolerance tests (GTT, ITT) in 22-week-old Nampt-AKO and Nampt-WT male mice fed a CD (n = 8 mice). **i** Analysis of adipocyte cell sizes in the brown adipose tissue (BAT), inguinal white adipose tissue (iWAT), and gonadal white adipose tissue (gWAT) of 18-week-old Nampt-AKO and Nampt-WT male mice fed a HFD for 4 weeks (n = 5 mice). **j** Quantitation of tyrosine hydroxylase (TH)$^{+}$ sympathetic nerve terminals in BAT, iWAT, and gWAT from 18-week-old Nampt-AKO and Nampt-WT male mice (n = 5 mice). arb. unit: arbitrary unit. Two-way repeated measures ANOVA followed by Fisher's LSD test (**a**, **d**, **e**, **f, g, h**) and two-sided unpaired t-test (**c**, **i**, **j**). n.s.; not significant. Two (**i**, **j**) or three (**a–h**) independent replicates were performed. Results are presented as mean ± SEM. Source data are provided as a Source Data file.

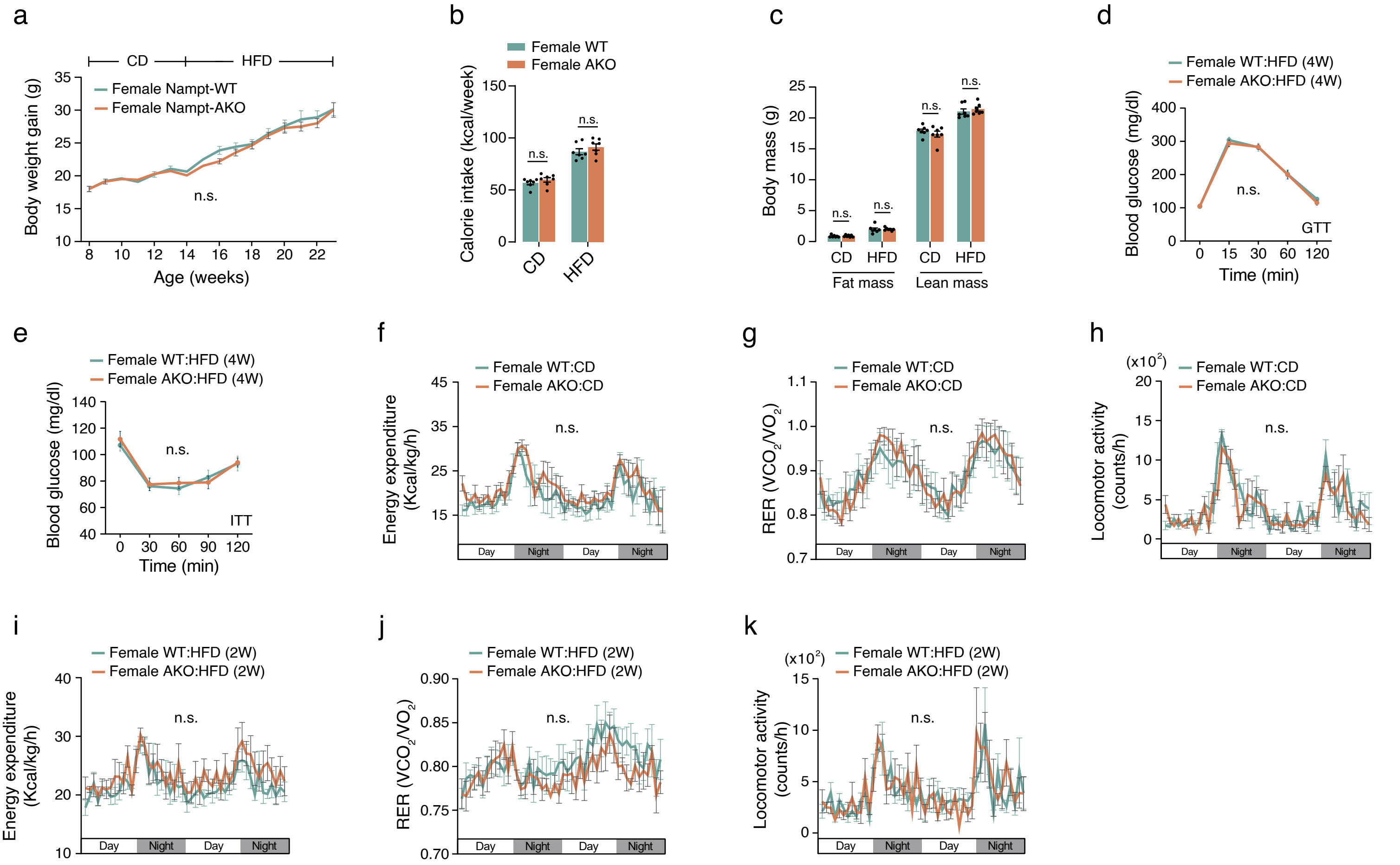

Supplementary Figure 3. Metabolic phenotype analysis in astrocyte-specific Nampt knockout female mice

a, **b** Comparison of body weights and calorie intakes between Nampt-AKO and Nampt-WT female mice following tamoxifen injection during sequential chow diet (CD) and high-fat diet (HFD) feeding conditions (n = 7 mice). **c** Body mass analysis in 18-week-old Nampt-AKO and Nampt-WT female mice (n = 7 mice). **d**, **e** Glucose and insulin tolerance tests (GTT, ITT) in 18-week-old Nampt-AKO and Nampt-WT female mice (n = 7 mice). **f–k** Energy expenditure, RER, and locomotor activity in 16-week-old Nampt-AKO and Nampt-WT female mice fed a CD (**f–h**) or HFD (**i–k**) (n = 4 mice). Two-way repeated measures ANOVA followed by Fisher's LSD test (**a**, **d–k**) and two-sided unpaired t-test (**b**, **c**). n.s.; not significant. Two independent replicates were performed for all studies. Results are presented as mean ± SEM. Source data are provided as a Source Data file.

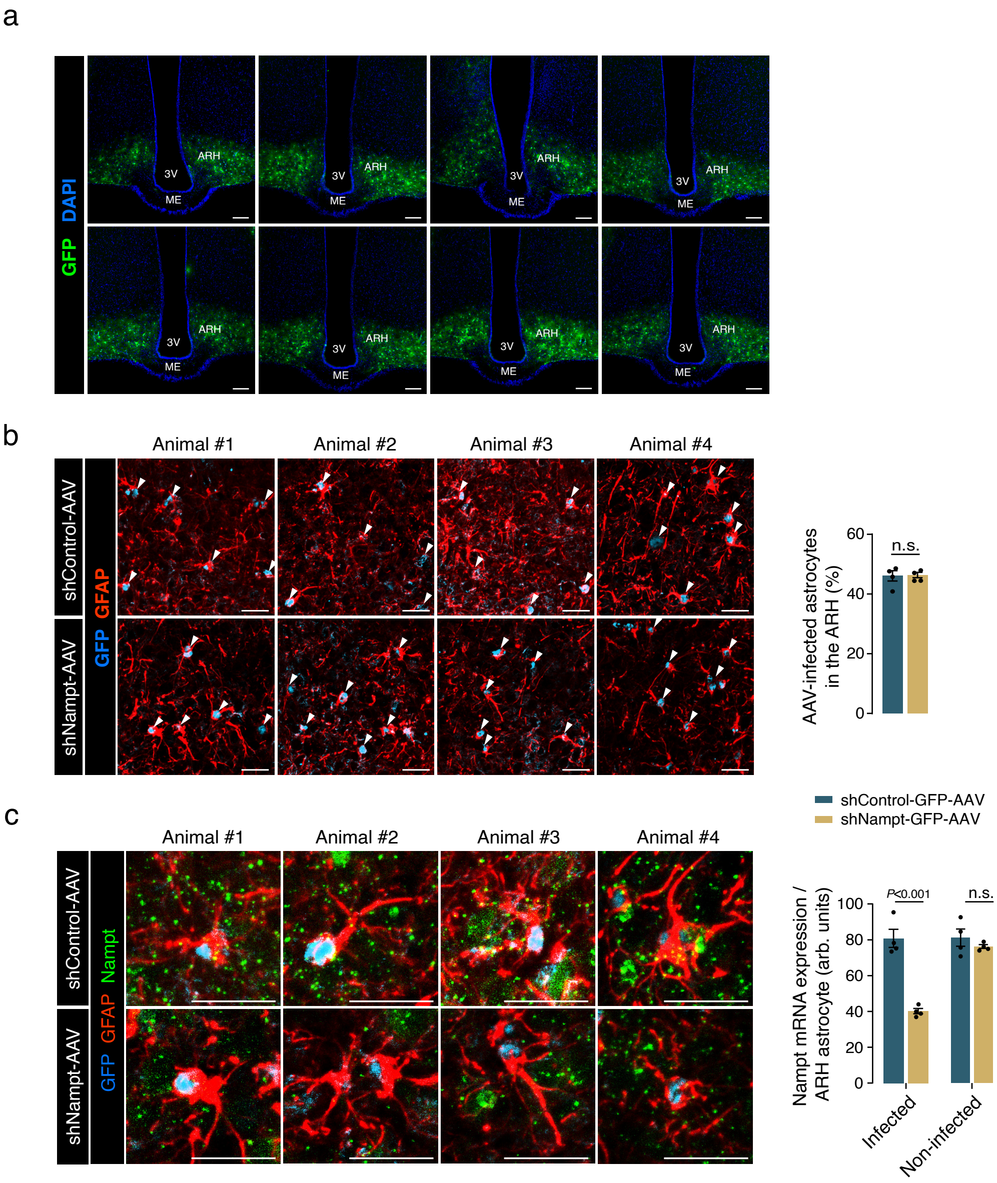

Supplementary Figure 4. Confirmation of successful viral infection of Nampt shRNA in the hypothalamic astrocytes

a Verification of successful viral injection by observing GFP expression restricted to the ARH. DIO-shNampt-eGFP-AAV or DIO-shControl-eGFP-AAV were injected into the bilateral ARH of 10-week-old hGFAP-Cre^{ERT2} male mice (n = 4 mice). Represented hypothalamic images are presented. Scale bars: 100 µm. 3V: thrid ventricle, ARH: hypothalamic arcuate nucleus, ME: median eminence. **b** Confirmation of successful viral infection in hypothalamic astrocytes by demonstrating GFP expression in GFAP^{+} ARH astrocytes in hGFAP-Cre^{ERT2} 16-week-old male mice injected with DIO-shNampt-eGFP-AAV or DIO-shControl-eGFP-AAV (n = 4 mice). Arrowheads indicate AAV-infected astrocytes. Scale bars: 25 µm. **c** Confirmation of Nampt knockdown in hypothalamic ARH astrocytes induced by injection of DIO-shNampt-AAV in 16-week-old hGFAP-Cre^{ERT2} male mice (n = 4 mice). Scale bars: 25 µm. arb. unit: arbitrary unit. Two-sided unpaired t-test (**b**, **c**). n.s.; not significant. Two independent replicates were performed for all studies.

Results are presented as mean ± SEM. Source data are provided as a Source Data file.

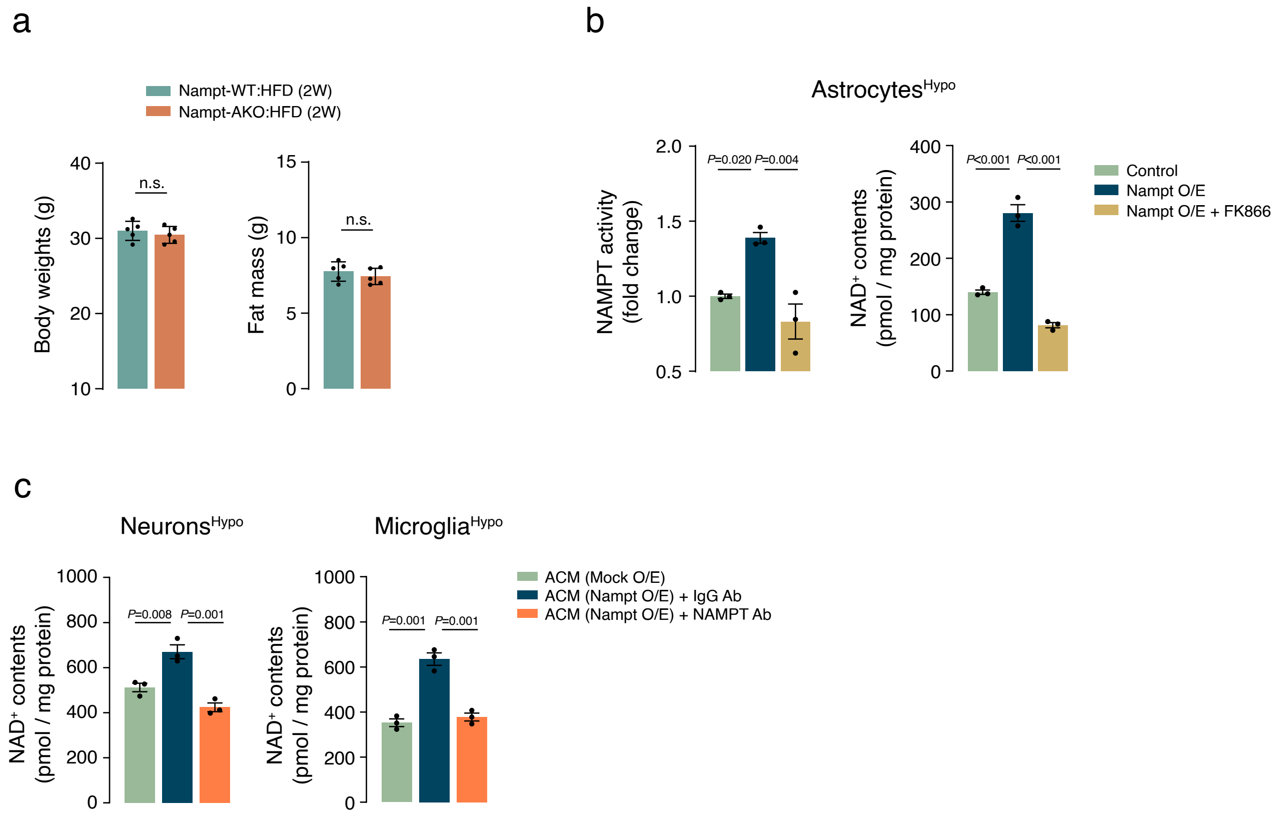

Supplementary Figure 5. Effects of NAMPT modulation on NAD^+ contents and NAMPT enzyme activity in hypothalamic astrocytes

a Body weights and fat mass of 16-week-old Nampt-AKO and Nampt-WT male mice that were subjected to hypothalamic leptin response study (n = 5 mice). **b** The changes in NAMPT activity and cellular NAD^+ contents in hypothalamic astrocytes subjected to Nampt overexpression (O/E) alone or with FK866 cotreatment (500 nM) (n = 3 wells). **c** Changes in cellular NAD^+ contents in primary cultured hypothalamic neurons and microglia treated with astrocyte conditioned medium (ACM) collected from astrocytes with or without Nampt O/E (n = 3 wells). Two-sided unpaired t-test (**a**) and one-way ANOVA followed by Fisher's LSD test (**b**, **c**). n.s.; not significant.

Two (**a**, **c**) or three (**b**) independent replicates were performed. Results are presented as mean ± SEM.

Source data are provided as a Source Data file.

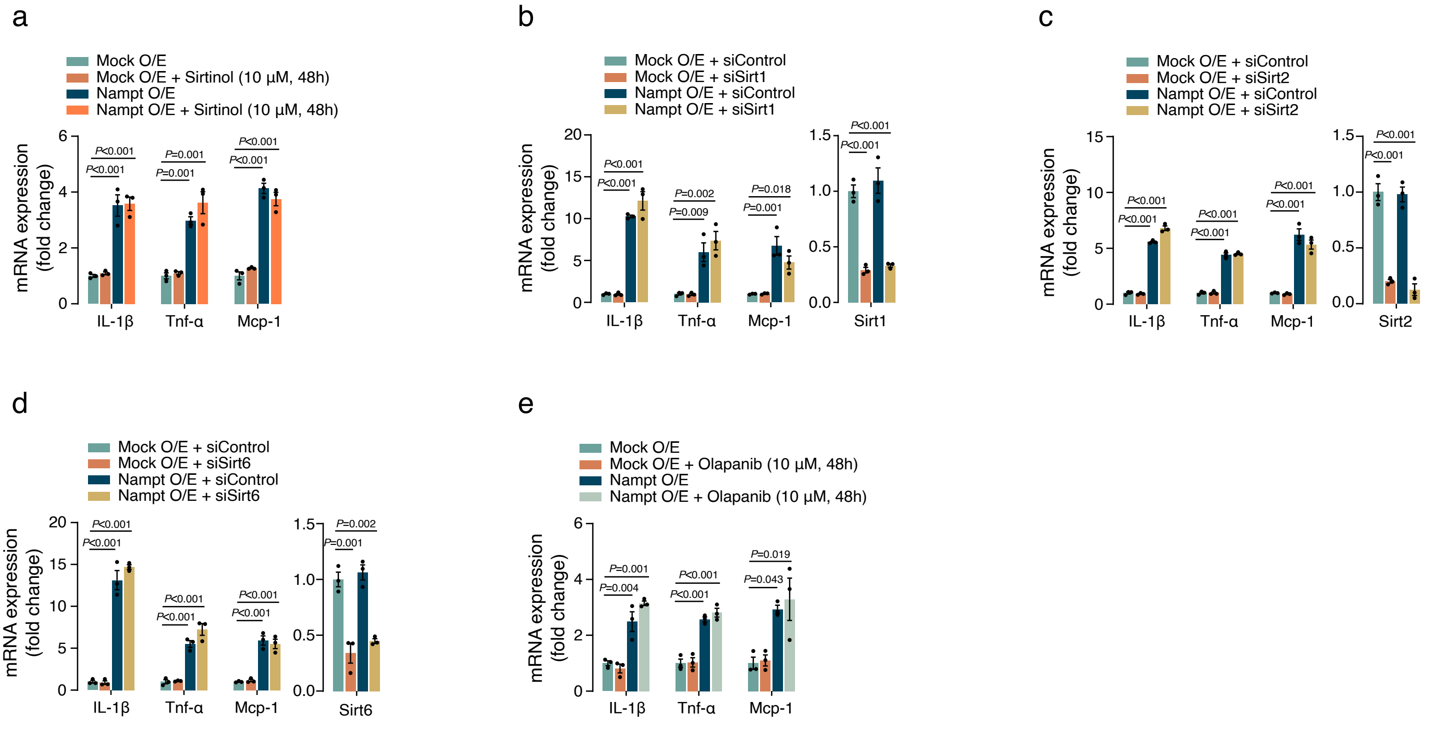

Supplementary Figure 6. Identification of the downstream mediator for the NAD⁺ salvage pathway-activated inflammation in hypothalamic astrocytes

Effects of SIRTs inhibition (**a–d**) and PARP inhibition (**e**) on Nampt overexpression (O/E)-induced inflammation in primary hypothalamic astrocytes (n = 3 wells). One-way ANOVA followed by Fisher's LSD test. Three independent replicates were performed for all studies. Results are presented as mean ± SEM. Source data are provided as a Source Data file.

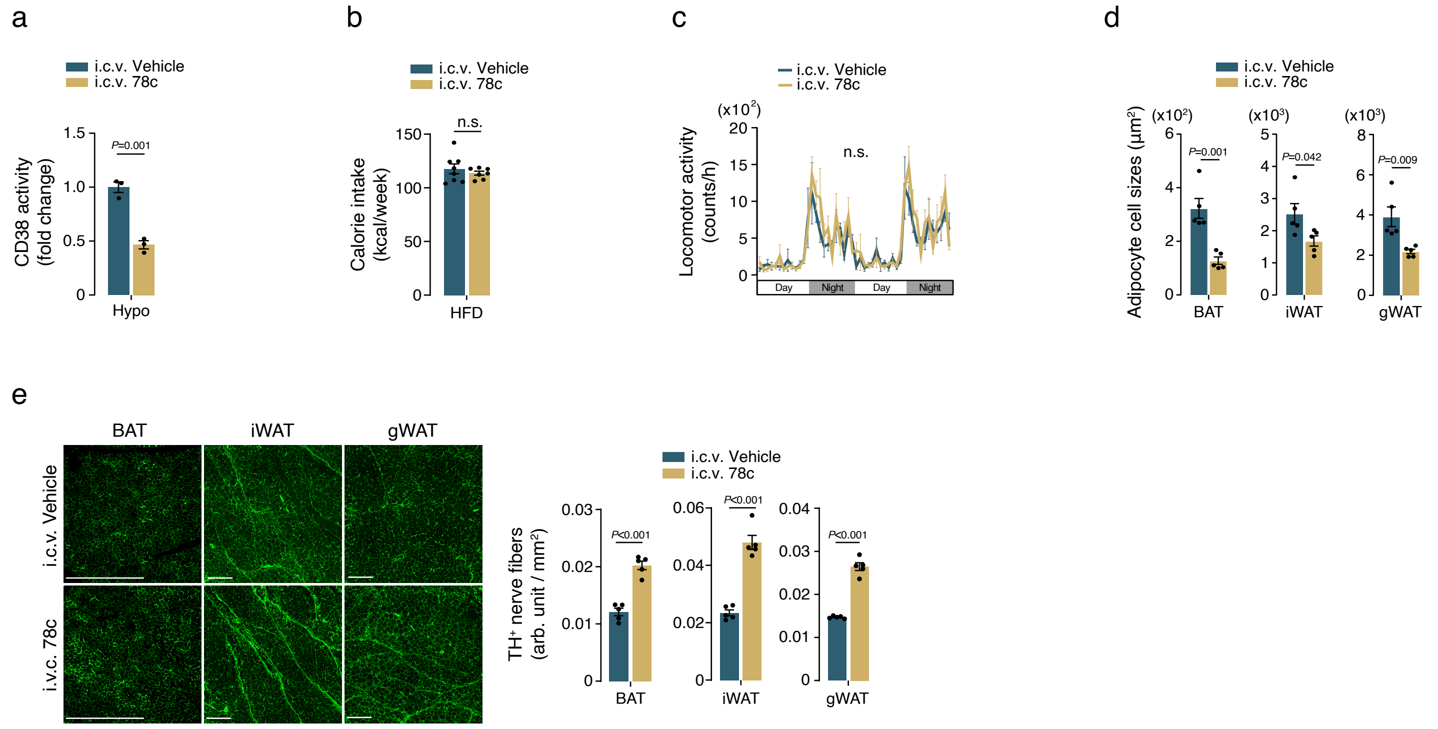

Supplementary Figure 7. Metabolic phenotype analysis in mice with i.c.v. injection of 78c

a Reduced hypothalamic CD38 enzyme activity in 16-week-old C57 male mice infused with 78c (n = 3 mice). **b**, **c** Calorie intakes (Vehicle, n = 8 mice; 78c, n = 7 mice) and locomotor activity (n = 5 mice) in 14~16 week-old C57 male mice during 78c treatment and HFD feding. **d**, **e** Adipocyte cell sizes analysis from H & E staining and sympathetic innervation in the BAT, iWAT, and gWAT of 16-week-old C57 male mice infused with 78c or vehicle and fed a HFD for 4 weeks (n = 5 mice). Scale bars: 500 μm. arb. unit: arbitrary unit. Two-sided unpaired t-test (**a**, **b**, **d**, **e**) and two-way repeated measures ANOVA followed by Fisher's LSD test (**c**). n.s.; not significant. Two (**a**, **d**, **e**) or three (**b**, **c**) independent replicates were performed. Results are presented as mean ± SEM.

Source data are provided as a Source Data file.

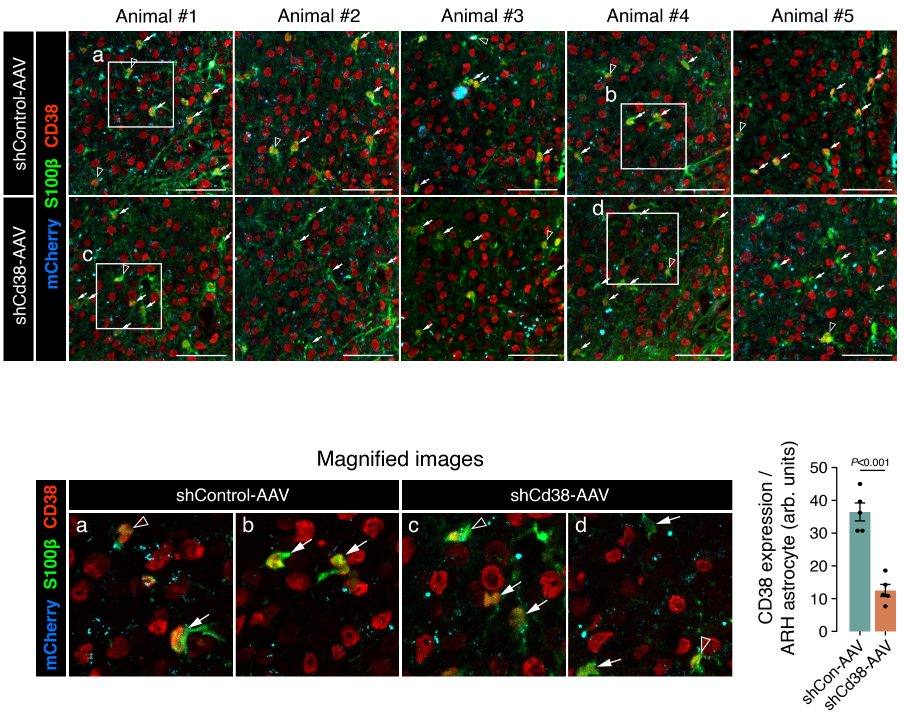

Supplementary Figure 8. Verification of successful Cd38 knockdown in the hypothalamic astrocytes

Confirmation of successful viral infection targeting Cd38 knockdown in hypothalamic astrocytes by demonstrating deletion of mCherry expression and reduced CD38 expression in hypothalamic ARH astrocytes in 16-week-old hGFAP-Cre^{ERT2} male mice (n = 5 mice). Arrows indicate soma of ARH astrocytes with successful viral infection, while arrowheads indicate soma of non-infected ARH astrocytes. Scale bars: 25 µm. arb. unit: arbitrary unit. Two-sided unpaired t-test. Two independent replicates were performed. Results are presented as mean ± SEM. Source data are provided as a Source Data file.

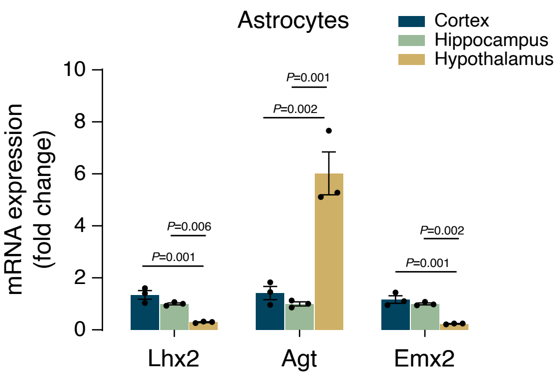

Supplementary Figure 9. Test of region specificity of astrocytes

To validate the accurate isolation of region-specific astrocytes, isolated astrocytes were subjected to qPCR analysis of brain region-specific astrocyte markers such as Lhx2, Agt, and Emx2 (n = 3 wells). One-way ANOVA followed by Fisher's LSD test. Two independent replicates were performed. Results are presented as mean ± SEM. Source data are provided as a Source Data file.

Supplementary Table 1. Primer sequences used in qPCR

Gene	Forward (5' → 3')	Reverse (5' → 3')
β-actin **(M)**	GGCTGTATTCCCCTCCATCG	CCAGTTGGTAACAATGCCATGT
Agt **(M)**	TCTCCTTTACCACAACAAGAGCA	CTTCTCATTCACAGGGGAGGT
C3 **(M)**	CCAGCTCCCCATTAGCTCTG	GCACTTGCCTCTTTAGGAAGTC
Cd38 **(M)**	TCTCTAGGAAAGCCCAGATCG	GTCCACACCAGGAGTGAGC
Emx2 **(M)**	TCAGCTACGCCAATTCCAGTC	ACCAAGTCCGGGTTGGAGTA
Gapdh **(M)**	AGGTCGGTGTGAACGGATTTG	TGTAGACCATGTAGTTGAGGTCA
Gapdh **(R)**	CTCATGACCACAGTCCATGC	TTCAGCTCTGGGATGACCTT
Gfap **(M)**	CGGAGACGCATCACCTCTG	AGGGAGTGGAGGAGTCATTCG
Iba1 **(M)**	ATCAACAAGCAATTCCTCGATGA	CAGCATTCGCTTCAAGGACATA
Il-1β **(M)**	GAAATGCCACCTTTTGACAGTG	TGGATGCTCTCATCAGGACAG
Il-1β **(R)**	CTGTGACTCGTGGGATGATG	GGGATTTTGTCGTTGCTTGT
Il-6 **(M)**	AGACTTCCATCCAGTTGCCT	CATTTCCACGATTTCCCAGAGA
Il-6 **(R)**	CCGGAGAGGAGACTTCACAG	ACAGTGCATCATCGCTGTTC
Il-10 **(M)**	CTTACTGACTGGCATGAGGATCA	GCAGCTCTAGGAGCATGTGG
Lhx2 **(M)**	CTGTTCCACAGTCTGTCGGG	CAGCAGGTAGTAGCGGTCAG
Nadsyn **(M)**	ACGGCTGCTCACTACTTGTTA	CTGAGAACCGAGGCAACTTC
Nampt **(M)**	ATCCAGGAGGCCAAAGAAGT	ATCGGGAGATGACCATCGTA
Naprt **(M)**	TGCTCACCGACCTCTATCAGG	CGAAGGAGCCTCCGAAAGG
Nmnat1 **(M)**	TGGCTCTTTTAACCCCATCAC	TCTTCTTGTACGCATCACCGA
Nmnat2 **(M)**	ATGACCGAGACCACAAAGACC	ATCCCGCCAATCACAATAAATCT
Nmnat3 **(M)**	CCTGTGGTTCCTTCAACCCC	AGATGATGCCCTCAATCACCT
Nrk1 **(M)**	TCATTGGAATTGGTGGTGTGAC	CAACAGGAAACTGCTGACATCAT
Nrk2 **(M)**	AAACTCATCATAGGCATTGGAGG	GTCCTGGGGCTTGAAGAAGT
Qaprt **(M)**	GGGGCTGACCTGGTAATGC	TGGGGAATCTGGCTTTAAGTGTA
S100β **(M)**	TGGTTGCCCTCATTGATGTCT	CCCATCCCCATCTTCGTCC
Sirt1 **(M)**	GTTCTGACTGGAGCTGGGGT	TCTGGGAGGTCTGGGAAGTC
Sirt2 **(M)**	CCGTCCACTGGCCTCTATGC	GGCAGATGGTTGGCTTGAAC

Sirt6 **(M)**	CTGAGAGACACCATTCTGGACT	GGTTGCAGGTTGACAATGACC
Tnfα **(M)**	GAGAAAGTCAACCTCCTCTCTG	GAAGACTCCTCCCAGGTATATG
Tnfα **(R)**	AGATGTGGAACTGGCAGAGG	CCCATTTGGGAACTTCTCCT
Mcp1 **(M)**	AGTAGGCTGGAGAGCTACAA	TGTCTGGACCCATTCCTTCT
Mcp1 **(R)**	ATGCAGTTAATGCCCCACTC	TTCCTTATTGGGGTCAGCAC
Mip2 **(M)**	CCAACCACCAGGCTACAGG	GCGTCACACTCAAGCTCTG
Mip2 **(R)**	AGGGTACAGGGGTTGTTGTG	TTTGGACGATCCTCTGAACC

M = Mouse, R = Rat
